# The extreme vertices of the power graph of a group

**DOI:** 10.1016/j.heliyon.2022.e12443

**Published:** 2022-12-15

**Authors:** Omar A. AbuGhneim, Mohammed Abudayah

**Affiliations:** a Department of Mathematics, The University of Jordan, Jordan; b School of Basic Sciences and Humanities, German Jordanian University, Jordan

**Keywords:** Power graph of a group, Extreme vertices, Abelian group, Dihedral group

## Abstract

For a fixed finite group *G*, the power graph of *G* was defined to be the simple graph Γ(G) whose vertex set V(Γ(G))=G, and edge set $E(\Gamma (G))=\{xy:\text{ either }x=y^{n}\text{ or }y=x^{n}\text{ for some integer }n\}$. In this paper the extreme vertices of the power graph of abelian groups, dihedral groups and dicyclic groups have been characterized.

## 1 Introduction

For a fixed finite group *G*, the directed power graph of *G*, $\overset{\to }{\Gamma }(G)$, was defined by Kelarev et al. (2001), to be the digraph whose vertex set is the elements of the finite group *G* and there is an arc from *x* to *y* if and only if x≠y and 〈y〉⊆〈x〉, that is $y=x^{m}$ for some integer *m*. Note here that digons (or bidirectional arcs) will appear if and only if 〈x〉=〈y〉. The underlying graph of power graph of *G* was first studied by Chakrabarty et al. (2009), it was denoted by Γ(G). To be more clear the underlying graph of the power graph of *G* is the graph with vertex set *G* and two different vertices x≠y are adjacent if and only if one of 〈x〉 and 〈y〉 is subset of the other.

Many researchers were attracted to work on both directed and undirected power graphs of a finite group *G*. For example Cameron and Ghosh (2011) proved that if power graphs of two finite groups *G* and *H* are isomorphic then *G* and *H* have the same number of elements of each order. Cameron (2010) answered the classical isomorphism question: For two abelian groups $G_{1}$ and $G_{2}$, if $\Gamma (G_{1})$ and $\Gamma (G_{1})$ are isomorphic, then $G_{1}$ and $G_{2}$ are isomorphic. Furthermore, they proved that for a power graph Γ(G) of a finite group *G*, the automorphism group is the same as that of its power graph if and only if *G* is the Klein 4-group. Also, Curtin and Pourgholi (2013) proved that maximum size of power graphs of finite groups, of same order, can be obtained in the set of the cyclic groups. In fact many graph invariants and properties of power graph and power digraph were also investigated, see Aalipour et al. (2017); Tamizh Chelvam and Sattanathan (2013); Mirzargar et al. (2012); Moghaddamfar et al. (2014); Pourgholi et al. (2015). In recent years, the study of power graphs has been growing for example, the strong metric dimension of the power graph of a finite group has been studied in Ma and Zhai (2021) and power graphs of (non)orientable genus two have been studied in Ma et al. (2019). Many results and open problems on power graphs can be found in the survay papers Abawajy et al. (2013) and Kumar et al. (2021).

Recall that if *G* is an abelian group of order *n* where $n=p_{1}^{k_{1}}p_{2}^{k_{2}}\ldots p_{m}^{k_{m}}$ is the prime decomposition of *n*, then $G≅G_{p_{1}}\times G_{p_{2}}\times \ldots G_{p_{m}}$ where $G_{p_{i}}=\{g\in G:|g|\text{ is a power of }p_{i}\}$. Finally, for two positive integers *r* and *m*, we denote greatest common divisor of *r* and *m* by gcd(r,m). Moreover the Euler's totient function of an integer *n*, ϕ(n), (some times called Euler's phi function) is defined to be the number of positive integers less than *n* and co-prime with *n*. For a graph *G*, the neighborhood of a vertex *v* is defined by $N_{G}(v)=\{u\in V(G):uv\in E(G)\}$, the degree of the vertex *v* is defined to be $d_{G}(v)=|N_{G}(v)|$. For any two vertices *u* and *v* of a connected graph *G*, d(u,v) denotes the length of a shortest path between *u* and *v*. Finally, a vertex *v* in a graph G is called an extreme vertex if the subgraph induced by its neighborhood is complete.

In this paper we investigate the extreme vertices of the power graph of a finite group. We find the extreme vertices of the power graph of finite abelian groups, dihedral groups and dicyclic groups. The abstract concept of convexity and extreme points concept were introduced and investigated in the fifties of last century. These concepts have been extended to graph theory. In fact extreme points play an essential role in studying abstract convexity especially in graph theory. For example every geodetic set of a graph must contain its extreme vertices.

## 2 Extreme vertices of power graph of groups

We examine the conditions on the elements of a group *G* to be extreme vertices of Γ(G).

Theorem 1 *Suppose that g is an element of a group G. If pq divides* |g| *, where p and q are two distinct primes, then g is not an extreme vertex of* Γ(G) *.*

Proof Observe that 〈g〉 contains an element of order *p* and an element of order *q*, say these elements are *x* and *y*. The two vertices *x* and *y* are adjacent to *g* in Γ(G) but they are not adjacent in Γ(G). Thus *g* is not an extreme vertex of Γ(G). □

According to the previous theorem, the candidates for extreme vertices in Γ(G) are elements in *G* of prime power order. We state this result in the following corollary.

Corollary 1 *Let g be an element of a group G. If g is an extreme vertex of* Γ(G) *, then* $|g|=p^{n}$ *where p is a prime number and n is a non-negative integer.*

In the following results, we examine some cases where elements of prime power order are not extreme vertices.

Theorem 2 *Let* p,q *and r be distinct prime numbers and g be an element of a group G with* $|g|=p^{i},i>0$ *. Suppose that G contains an element x of order* $p^{n}q$ *and an element y of order* $p^{n}r$ *with* g∈〈x〉 *and* g∈〈y〉 *. Then g is not an extreme vertex of* Γ(G) *.*

Proof Since g∈〈x〉 and g∈〈y〉, then the vertex *g* is adjacent to both of the vertices *x* and *y* in Γ(G). Since $|x|=p^{n}q$, $|y|=p^{n}r$ and *q* and *r* are distinct primes, then the vertices *x* and *y* are not adjacent in Γ(G). Therefore *g* is not an extreme vertex of Γ(G). □

Theorem 3 *Let p and q be two distinct prime numbers and g be an element of a group G with* $|g|=p^{i},i>0$ *. Suppose that G contains an element x of order* $p^{n}$ *,* n>i *, and an element y of order* $p^{m}q$ *,* i≤m<n *, with* g∈〈x〉 *and* g∈〈y〉 *. Then g is not an extreme vertex of* Γ(G) *.*

Proof It is similar to the proof of Theorem 2. □

Theorem 4 *Let* p,q *and r be distinct prime numbers and g be an element of a group G with* $|g|=p^{i},i>0$ *. Suppose that G contains an element x of order* $p^{n}qr$ *such that* g∈〈x〉 *. Then g is not an extreme vertex of* Γ(G) *.*

Proof Since g∈〈x〉, then the vertex *g* is adjacent to *x* in Γ(G). We have $|x^{q}|=p^{n}r$ and $|x^{r}|=p^{n}q$. Note that the two vertices $x^{q}$ and $x^{r}$ are adjacent to the vertex *g* but they are not adjacent to each other in Γ(G). Therefore *g* is not an extreme vertex of Γ(G). □

## 3 Extreme vertices of power graphs of Abelian groups

Now, let us look at the extreme vertices of Γ(G) where *G* is an abelian group. The following theorem shows that Γ(G) has no extreme vertices for many abelian groups.

Theorem 5 *Let p, q and r be distinct prime numbers and G be an abelian group. If pqr divides the order of G then* Γ(G) *has no extreme vertices.*

Proof According to Corollary 1, an element x∈G is a possible extreme vertex if its order is of prime power. Suppose that x∈G and $|x|=p_{1}^{n}$ where $p_{1}$ is a prime number. The prime number $p_{1}$ is not equal to at least two of the primes *p*, *q* and *r*. Without loss of generality we can assume that $p_{1}\neq q$ and $p_{1}\neq r$. Since *q* and *r* are primes, then *G* has an element of order *q* and an element of order *r*, say these elements are *y* and *z*, respectively. Since *G* is abelian, then *xy* and *xz* are elements of order $p_{1}^{n}q$ and $p_{1}^{n}r$, respectively. Using Theorem 2, *x* is not an extreme vertex of Γ(G) and thus Γ(G) has no extreme vertices. □

Let *G* be an abelian group. According to previous theorem Γ(G) can have extreme vertices only if $|G|=p^{n}q^{m}$ where *p* and *q* are prime numbers. We have the following results for cyclic groups of order $p^{n}q^{m}$.

Theorem 6 *Let p and q be two distinct prime numbers and G be a cyclic group of order* $p^{n}q^{m}$ *where n and m are positive integers. If* x∈G *and* $|x|=p^{i}$ *where* i<n *, then x is not an extreme vertex of* Γ(G) *.*

Proof Suppose that x∈G and $|x|=p^{i}$ where i<n. Then $x=a^{p^{n-i}}$ where *a* is an element of order $p^{n}$. Consider the two elements y=a and $z=a^{p^{n-i}}b$ where *b* is an element of order *q* in *G*. Since gcd$(q,p^{n})=1$, then there exists a positive integer *t* such tq≡1 modulo $p^{n}$. Therefore, there exists an integer *s* such that $sp^{n}+1=tq$. Now $z^{tq}={\left(a^{p^{n-i}}b\right)}^{tq}={\left(a^{p^{n-i}}\right)}^{tq}\;b^{tq}={\left(a^{p^{n-i}}\right)}^{tq}$, since |b|=q. Because $sp^{n}+1=tq$, then $z^{tq}={\left(a^{p^{n-i}}\right)}^{sp^{n}+1}={\left(a^{sp^{n}}a^{1}\right)}^{p^{n-i}}=a^{p^{n-i}}$. The last equality follows from the fact that $|a|=p^{n}$. Hence $x=a^{p^{n-i}}\in \langle a^{p^{n-i}}b\rangle =\langle z\rangle$. Also x∈〈y〉=〈a〉. Use Theorem 3 to get *x* is not an extreme vertex of Γ(G). □

Theorem 7 *Let p and q be two distinct prime numbers and G be a cyclic group of order* $p^{n}q^{m}$ *where n and m are positive integers. If* x∈G *and* $|x|=p^{n}$ *, then x is an extreme vertex of* Γ(G) *.*

Proof Suppose that x∈G and $|x|=p^{n}$. Since *G* is a cyclic group of order $p^{n}q^{m}$, then G=HK where *H* is a cyclic group of order $p^{n}$ and *K* is a cyclic group of order $q^{m}$ with H∩K={e} (*G* is the internal direct product of *H* and *K*). Since $\left|x|=p^{n}=|H\right|$, then H=〈x〉 and the vertex *x* is adjacent to all the elements of H=〈x〉, i.e. *x* is adjacent to all elements of order $p^{i},i\leq n$. These are all the elements *z* such that |z| divides |x| and it is clear that all of these elements are mutually adjacent. If *y* is another element that is adjacent to *x*, then |x| divides |y| and thus $|y|=p^{n}q^{j}$ for some j>0. Since *G* is cyclic and $|y|=p^{n}q^{j}$, then 〈x〉⊆〈y〉 and thus *x* is adjacent to *y*. Therefore the vertices that are adjacent to *x* are all the elements of order $p^{i}$ where 0≤i≤n and elements of order $p^{n}q^{j}$ where 1≤j≤m. It is clear that elements of order $p^{i}$ where 0≤i≤n are adjacent to each other. It is easy to check that elements of order $p^{i}$ where 0≤i≤n are adjacent to elements of order $p^{n}q^{j}$ where 1≤j≤m. Suppose that $y_{1}$ and $y_{2}$ are elements of order $p^{n}q^{j_{1}}$ and $p^{n}q^{j_{2}}$, respectively with $j_{1}\leq j_{2}$. Since *G* is cyclic, then $\langle y_{1}\rangle \subseteq \langle y_{2}\rangle$ and so $y_{1}$ and $y_{2}$ are adjacent in Γ(G). Hence *x* is an extreme vertex of Γ(G). □

We get the same results for elements of order $q^{j}$ where j≤m. Combining these results with Theorem 1 we get the following corollary.

Corollary 2 *Let p and q be two distinct prime numbers and G be a cyclic group of order*$p^{n}q^{m}$*where n and m are positive integers. An element*x∈G*is an extreme vertex of*Γ(G)*if and only if*$|x|=p^{n}$*or*$q^{m}$*.*

According to Theorem 5, Theorem 6 and Theorem 7 we get the following classification for the extreme vertices of the power graph of a cyclic group.

Corollary 3 *Let G be a cyclic group and p, q are prime numbers.*

• *If*|G|*has three distinict prime factors, then*Γ(G)*has no extreme vertices.*
• *If*$|G|=p^{n}q^{m}$*, say*G=〈x〉*and*$|x|=p^{n}q^{m}$*, then the set of extreme vertices of*Γ(G)*is*
  $$\{x^{ip^{n}}:gcd(i,p^{n}q^{m})=1\}\cup \{x^{jq^{m}}:gcd(j,p^{n}q^{m})=1\}.$$
• *If*$|G|=p^{n}$*, then*Γ(G)*is complete graph, see**Chakrabarty et al. (2009)**. Thus the extreme vertices of*Γ(G)*are the set of all elements of G.*

Now, we want to look at abelian but not cyclic groups of order $p^{n}q^{m}$. Assume *G* is an abelian group of order $p^{n}q^{m}$. Then $G≅\langle x_{1}\rangle \times \langle x_{2}\rangle \times \ldots \times \langle x_{r}\rangle \times \langle y_{1}\rangle \times \langle y_{2}\rangle \times \ldots \times \langle y_{s}\rangle$, where $|x_{i}|=p^{n_{i}}$, $|y_{j}|=q^{m_{j}}$, 1≤i≤r, 1≤j≤s, $1\leq n_{1}\leq n_{2}\leq \ldots \leq n_{r}$ and $1\leq m_{1}\leq m_{2}\leq \ldots \leq m_{s}$. If g∈G, then $g=x_{1}^{\alpha_{1}}x_{2}^{\alpha_{2}}\ldots x_{r}^{\alpha_{r}}y_{1}^{\beta_{1}}y_{2}^{\beta_{2}}\ldots y_{s}^{\beta_{s}}$, where the $\alpha_{i}$'s ≥0 and the $\beta_{i}$'s ≥0. If one of the $\alpha_{i}$'s >0 and one of the $\beta_{i}$'s >0, then *pq* divides the order of *g* and according to Theorem 1 *g* is not an extreme vertex of Γ(G).

Assume $g=x_{1}^{\alpha_{1}}x_{2}^{\alpha_{2}}\ldots x_{r}^{\alpha_{r}}$ and r≥2. Then $y_{1}\neq e$, $y_{2}\neq e$, $|y_{1}^{q^{m_{1}-1}}|=q$ and $|y_{2}^{q^{m_{2}-1}}|=q$. Consider $z_{1}=gy_{1}^{q^{m_{1}-1}}$ and $z_{2}=gy_{2}^{q^{m_{2}-1}}$. Then $z_{1}^{q}=g^{q}$ and since *p* and *q* are relatively prime, we get $\left|g|=|g^{q}\right|$. Thus $\langle g\rangle =\langle g^{q}\rangle \subseteq \langle z_{1}\rangle$ and so *g* and $z_{1}$ are adjacent in Γ(G). Similarly, *g* and $z_{2}$ are adjacent in Γ(G). Since $z_{1}\notin \langle z_{2}\rangle$ and $z_{2}\notin \langle z_{1}\rangle$, then $z_{1}$ and $z_{2}$ are not adjacent in Γ(G). Hence *g* is not an extreme vertex of Γ(G). According to this we get the following theorem.

Theorem 8 *Suppose that G is an abelian group with* $G≅\langle x_{1}\rangle \times \langle x_{2}\rangle \times \ldots \times \langle x_{r}\rangle \times \langle y_{1}\rangle \times \langle y_{2}\rangle \times \ldots \times \langle y_{s}\rangle$ *, where* $|x_{i}|=p^{n_{i}}$ *,* $|y_{j}|=p^{n_{j}}$ *,* 1≤i≤r *and* 1≤j≤s *(* $|G|=p^{n}q^{m}$ *). If* r,s≥2 *, then* Γ(G) *has no extreme vertices.*

Now, we want to look at abelian groups of the form $G≅\langle x_{1}\rangle \times \langle x_{2}\rangle \times \ldots \times \langle x_{r}\rangle \times \langle y_{1}\rangle$, where $|x_{i}|=p^{n_{i}}$, 1≤i≤r, $|y_{1}|=q^{m}$, $1\leq n_{1}\leq n_{2}\leq \ldots \leq n_{r}$, m≥1 and r≥2 (case r=1 gives cyclic groups and we have discussed cyclic groups before). Suppose that g∈G and *g* is of the form $g=x_{1}^{\alpha_{1}}x_{2}^{\alpha_{2}}\ldots x_{r}^{\alpha_{r}}y_{1}^{\beta_{1}}$, where the $\alpha_{i}$'s ≥0 and $0<\beta_{1}<|y_{1}|$. If one of the $\alpha_{i}$'s >0, then using Theorem 1, *g* is not an extreme vertex of Γ(G). If all $\alpha_{i}$'s =0, then $g=y^{p_{1}}$ and using a similar argument to the one before Theorem 8, we get *g* is not an extreme vertex of Γ(G). Thus candidates for extreme vertices are elements of the form $g=x_{1}^{\alpha_{1}}x_{2}^{\alpha_{2}}\ldots x_{r}^{\alpha_{r}}$, where the $\alpha_{i}$'s ≥0. In the following theorems, we characterize which ones of these elements are extreme vertices.

Theorem 9 *Suppose that G is an abelian group and* $G≅\langle x_{1}\rangle \times \langle x_{2}\rangle \times \ldots \langle x_{r}\rangle \times \langle y_{1}\rangle$ *, where* $|x_{i}|=p^{n_{i}}$ *,* 1≤i≤r *,* $|y_{1}|=q^{m}$ *,* $1\leq n_{1}\leq n_{2}\leq \ldots \leq n_{r}$ *,* m≥1 *and* r≥2 *. Let* $g=x_{1}^{\alpha_{1}}x_{2}^{\alpha_{2}}\ldots x_{r}^{\alpha_{r}}$ *and* $|x_{i}^{\alpha_{i}}|=p^{t_{i}}$ *such that* $p^{t_{i}}<p^{n_{i}}$ *(* $t_{i}<n_{i}$ *) for all i. Then g is not an extreme vertex of* Γ(G) *.*

Proof Since $\left|x_{i}^{\alpha_{i}}|=p^{t_{i}}<p^{n_{i}}=|x_{i}\right|$, one can find $z_{i}\in \langle x_{i}\rangle$ such that $z_{i}^{p}=x_{i}^{\alpha_{i}}$ for all *i*. Define $z=z_{1}z_{2}\ldots z_{r}$. Observe that if $|g|=p^{l}$, then $|z|=p^{l+1}$ and $z^{p}=z_{1}^{p}z_{2}^{p}\ldots z_{r}^{p}=x_{1}^{\alpha_{1}}x_{2}^{\alpha_{2}}\ldots x_{r}^{\alpha_{r}}$. Thus *g* is adjacent to *z* in Γ(G). Now, define w=gy where $y\in \langle y_{1}\rangle$ and |y|=q. Observe that $w^{q}=g^{q}$. Since *p* and *q* are relatively primes, then $\left|g^{q}|=|g\right|$ and thus $\langle g\rangle =\langle g^{q}\rangle \subseteq \langle w\rangle$. Hence *g* is adjacent to *w* in Γ(G). So, we have seen that *g* is adjacent to *z* with $|z|=p^{l+1}$ and it is also adjacent to *w* with $|w|=p^{l}q$. Since |z| does not divide |w| and |w| does not divide |z|, then *z* and *w* are not adjacent in Γ(G). Therefore *g* is not an extreme vertex of Γ(G). □

The following example explains previous theorem.

Example 1 Let $G=Z_{3^{2}}\times Z_{3^{3}}\times Z_{5^{2}}$. Then $g=(3,3^{2},0)$ is not an extreme vertex of Γ(G). Observe that z=(1,3,0) and $w=(3,3^{2},5)$ are adjacent to *g* but *z* and *w* are not adjacent in Γ(G).

Suppose that *G* is an abelian group and $G≅\langle x_{1}\rangle \times \langle x_{2}\rangle \times \ldots \langle x_{r}\rangle \times \langle y_{1}\rangle$, where $|x_{i}|=p^{n_{i}}$, 1≤i≤r, $|y_{1}|=q^{m}$, $1\leq n_{1}\leq n_{2}\leq \ldots \leq n_{r}$, m≥1 and r≥2. According to previous theorem, the only candidates for extreme vertices in Γ(G) are elements of the form $g=x_{1}^{\alpha_{1}}x_{2}^{\alpha_{2}}\ldots x_{r}^{\alpha_{r}}$ such that $|x_{i}^{\alpha_{i}}|=p^{t_{i}}$ and $t_{i}=n_{i}$ for at least one *i*. Ultimately, we will show that these elements are the extreme vertices of Γ(G). First, we want to find all the elements that are adjacent to *g* where $g=x_{1}^{\alpha_{1}}x_{2}^{\alpha_{2}}\ldots x_{r}^{\alpha_{r}}$ such that $|x_{i}^{\alpha_{i}}|=p^{t_{i}}$ and $t_{i}=n_{i}$ for at least one *i*. To do that we need the following notations and results from Sehgal and Singh (2019). We write $d_{G}^{+}(g)$, $d_{G}^{-}(g)$ and $d_{G}^{\pm }(g)$ to denote respectively the out-degree of *g*, the in-degree of *g* and the number of bidirectional edges incident to *g* in the diagraph $\overset{\to }{\Gamma }(G)$. Note that the degree of a vertex *g* in Γ(G) equals the sum of the in-degree and out-degree of *g* minus the number of bidirectional edges incident to *g*. It is easy to check that $d_{G}^{+}(g)=|\langle g\rangle |-1=|g|-1$ and $d_{G}^{\pm }(g)=\phi (|g|)-1$. Thus $d_{G}(g)=|g|-\phi (|g|)+d_{G}^{-}(g)$. To determine $d_{G}(g)$, we need to count $d_{G}^{-}(g)$. In Sehgal and Singh (2019), the authors investigated this problem for abelian groups and gave the following results.

Theorem 10 *(* *Sehgal and Singh (2019)* *) Let* $G=\langle x_{1}\rangle \times \langle x_{2}\rangle \times \ldots \times \langle x_{r}\rangle$ *be an abelian p-group where* $|x_{i}|=p^{n_{i}}$ *,* 1≤i≤r *and* $1\leq n_{1}\leq n_{2}\leq \ldots \leq n_{r}$ *. If* $g=\prod_{\alpha =1}^{r}\;x_{\alpha }^{i_{\alpha }}$ *is a nonidentity element of G and* $|x_{\alpha }^{i_{\alpha }}|=p^{t_{\alpha }}$ *, then*

$$d_{G}^{-}(g)=-1+\phi (|g|)\;\;\sum_{\beta =0}^{\;\text{min}\{n_{k+1}-t_{k+1},\ldots ,n_{r}-t_{r}\}}\;\;p^{\sum_{j=1}^{r}\;\text{min}\{n_{j},\beta \}},$$

*where k is the smallest non-negative integer such that* $|x_{k+1}^{i_{k+1}}|\neq 1$ *.*

Theorem 11 *(* *Sehgal and Singh (2019)* *) Let G be a group and let H and K be two normal subgroups of G such that* |H| *and* |K| *are relatively prime. If G is the internal direct product of the subgroups H and K, then for an element* z=xy *of the group G where* x∈H *and* y∈K *,*

i $d_{G}^{+}(z)=\left(d_{H}^{+}(x)+1\right)\left(d_{K}^{+}(y)+1\right)-1$ *.*
ii $d_{G}^{\pm }(z)=\left(d_{H}^{\pm }(x)+1\right)\left(d_{K}^{\pm }(y)+1\right)-1$ *.*
iii $d_{G}^{-}(z)=\left(d_{H}^{-}(x)+1\right)\left(d_{K}^{-}(y)+1\right)-1$ *.*

As mentioned earlier, our objective is to find all the elements that are adjacent to *g* where $g=x_{1}^{\alpha_{1}}x_{2}^{\alpha_{2}}\ldots x_{r}^{\alpha_{r}}$ such that $|x_{i}^{\alpha_{i}}|=p^{t_{i}}$ ($t_{i}\leq n_{i}$) and $t_{i}=n_{i}$ for at least one *i*. The element *g* is in the abelian group $G=\langle x_{1}\rangle \times \langle x_{2}\rangle \times \ldots \times \langle x_{r}\rangle \times \langle y_{1}\rangle$, where $|x_{i}|=p^{n_{i}}$, 1≤i≤r, $|y_{1}|=q^{m}$, $1\leq n_{1}\leq n_{2}\leq \ldots \leq n_{r}$, m≥1 and r≥2. Let $H=\langle x_{1}\rangle \times \langle x_{2}\rangle \times \ldots \times \langle x_{r}\rangle$ and $K=\langle y_{1}\rangle$. Observe that $t_{i}=n_{i}$ for at least one *i*, and thus we get

$$\sum_{\beta =0}^{\text{ min}\{n_{k+1}-t_{k+1},\ldots ,n_{r}-t_{r}\}}\;\;p^{\sum_{j=1}^{r}\text{ min}\{n_{j},\beta \}}=1.$$

Therefore, according to Theorem 10 we get $d_{H}^{-}(g)=\phi (|g|)-1$ and thus $d_{H}(g)=|g|-\phi (|g|)+d_{H}^{-}(g)=|g|-1$. Write g=g⋅1 where g∈H and 1∈K. Use Theorem 11 to get

$d_{G}^{+}(g)=\left(d_{H}^{+}(g)+1\right)\left(deg_{K}^{+}(1)+1\right)-1=(|g|-1+1)(0+1)-1=|g|-1$,

$d_{G}^{\pm }(g)=\left(d_{H}^{\pm }(g)+1\right)\left(d_{K}^{\pm }(1)+1\right)-1=(\phi (|g|)-1+1)(0+1)-1=\phi (|g|)-1$ and

$d_{G}^{-}(g)=\left(d_{H}^{-}(g)+1\right)\left(d_{K}^{-}(1)+1\right)-1=(\phi (|g|)-1+1)(|K|-1+1)-1=\phi (|g|)|K|-1$. Thus $d_{G}(g)=(|g|-1)+(\phi (|g|)|K|-1)-(\phi (|g|)-1)=\phi (|g|)(|K|-1)+|g|-1$.

Consider the sets A=〈g〉−{g} and $B=\{g^{a}y_{1}^{b}:\text{gcd}(a,p)=1\text{ and }1\leq b\leq q^{m}-1\}$. It is easy to check that |A∪B|=(|g|−1)+ϕ(|g|)(|K|−1) and each element of A∪B is adjacent to *g*. Since $d_{G}(g)=|A\cup B|$ and each element of A∪B is adjacent to *g*, then the elements of A∪B are precisely the vertices that are adjacent to *g* in Γ(G). We are now in a position to determine the extreme vertices of Γ(G).

Theorem 12 *Suppose that G is an abelian group and* $G≅\langle x_{1}\rangle \times \langle x_{2}\rangle \times \ldots \langle x_{r}\rangle \times \langle y_{1}\rangle$ *, where* $|x_{i}|=p^{n_{i}}$ *,* 1≤i≤r *,* $|y_{1}|=q^{m}$ *,* $1\leq n_{1}\leq n_{2}\leq \ldots \leq n_{r}$ *,* m≥1 *and* r≥2 *. The extreme vertices of* Γ(G) *are precisely the elements g of the form* $g=x_{1}^{\alpha_{1}}x_{2}^{\alpha_{2}}\ldots x_{r}^{\alpha_{r}}$ *such that* $|x_{i}^{\alpha_{i}}|=p^{t_{i}}$ *and* $t_{i}=n_{i}$ *for at least one i.*

Proof According to what we have shown prior to Theorem 10, the only candidates for extreme vertices in Γ(G) are elements of the form $g=x_{1}^{\alpha_{1}}x_{2}^{\alpha_{2}}\ldots x_{r}^{\alpha_{r}}$ such that $|x_{i}^{\alpha_{i}}|=p^{t_{i}}$ and $t_{i}=n_{i}$ for at least one *i*. Our goal is to show that any element of this form is an extreme vertex of Γ(G). We have seen that the elements that are adjacent to *g* are precisely the elements of the set A∪B where A=〈g〉−{g} and $B=\{g^{a}y_{1}^{b}:\text{gcd}(a,p)=1\text{ and }1\leq b\leq q^{m}-1\}$. The induced subgraph on 〈g〉 forms a complete graph because $|g|=p^{l}$ for some l≥1. Let $g^{a}y^{b}\in B$. Then ${\left(g^{a}y_{1}^{b}\right)}^{q^{m}}=g^{aq^{m}}y_{1}^{bq^{m}}=g^{aq^{m}}$ and since gcd$(aq^{m},p)=1$, we get $\langle g\rangle =\langle g^{aq^{m}}\rangle \subseteq \langle g^{a}y_{1}^{b}\rangle$. Thus any element of *A* is adjacent to all the elements of *B*. Let $g^{a_{1}}y_{1}^{b_{1}},g^{a_{2}}y_{1}^{b_{2}}\in B$ with $\left|y_{1}^{b_{1}}|=q^{m1}\leq q^{m2}=|y_{1}^{b_{2}}\right|$. Then $g^{a_{1}}y_{1}^{b_{1}}\in \langle g^{a_{2}}y_{1}^{b_{2}}\rangle$ and thus any element of *B* is adjacent to any other element of *B*. Therefore, the induced subgraph on A∪B is complete and hence *g* is an extreme vertex of Γ(G). □

For instance, the elements $\left(1,3,0),(2,3^{2},0\right)$ and (3,1,0) are extreme vertices in $G=Z_{3^{2}}\times Z_{3^{3}}\times Z_{5^{2}}$.

Using a similar argument, we get the following result.

Theorem 13 *Suppose that G is an abelian p-group where* $G≅\langle x_{1}\rangle \times \langle x_{2}\rangle \times \ldots \times \langle x_{r}\rangle$ *and* r≥2 *. The extreme vertices of* Γ(G) *are precisely the elements g of the form* $g=x_{1}^{\alpha_{1}}x_{2}^{\alpha_{2}}\ldots x_{r}^{\alpha_{r}}$ *such that* $|x_{i}^{\alpha_{i}}|=p^{t_{i}}$ *and* $t_{i}=n_{i}$ *for at least one i.*

## 4 Extreme vertices of power graphs of dihedral and dicyclic groups

In this section, we examine the extreme vertices for the dihedral and dicyclic groups. The dihedral group of order 2*n* is

$$D_{2n}=\langle x,y:x^{n}=y^{2}=1\text{ and }yxy=x^{-1}\rangle .$$

Chattopadhyay and Panigrahi (2014) characterized power graphs of dihedral groups.

Theorem 14 *(* *Chattopadhyay and Panigrahi (2014)* *) For* n≥3 *,* $\Gamma (D_{2n})≅\left(\Gamma (Z_{n}-\{0\})\right)\cup nK_{1}+K_{1}$ *.*

The vertices of $nK_{1}$ in $\Gamma (D_{2n})$ are $y,xy,x^{2}y,\ldots ,x^{n-1}y$. These vertices are of degree one in $\Gamma (D_{2n})$ and thus each one of them is an extreme vertex of $\Gamma (D_{2n})$. Since $\Gamma (D_{2n})≅\Gamma (Z_{n}-\{0\})\cup nK_{1}+K_{1}$, then the other extreme vertices of $\Gamma (D_{2n})$ are the extreme vertices of $\Gamma (Z_{n})$. Use Theorem 5 and Corollary 2 to get the following result about the extreme vertices of $\Gamma (D_{2n})$.

Theorem 15 *The extreme vertices of* $\Gamma (D_{2n})$ *are* $y,xy,x^{2}y,\ldots ,x^{n-1}y$ *if and only if n has three different prime factors. If* $n=p^{m}q^{k}$ *where p and q are distinict primes, then the extreme vertices of* $\Gamma (D_{2n})$ *are* $y,xy,x^{2}y,\ldots ,x^{n-1}y$ *, all elements of* $D_{2n}$ *of order* $p^{m}$ *and all elements of* $D_{2n}$ *of order* $q^{k}$ *. If* $n=p^{m}$ *where p is a prime number, then the extreme vertices of* $\Gamma (D_{2n})$ *are all elements of* $D_{2n}$ *except the identity element.*

The dicyclic group of order 4*n* is

$$Q_{4n}=\langle x,y:x^{2n}=y^{4}=1,x^{n}=y^{2}\text{ and }yx=x^{-1}y\rangle .$$

The characterization of the power graphs of dicyclic groups was given in Chattopadhyay and Panigrahi (2014).

Theorem 16 *(* *Chattopadhyay and Panigrahi (2014)* *) For* n≥2 *,* $\Gamma (Q_{4n})≅\Gamma (\langle x\rangle )\cup n(A+K_{2})$ *where A is the subgraph of* Γ(〈x〉) *induced by* $\left\{1,x^{n}\right\}$ *.*

**Figure 1:**
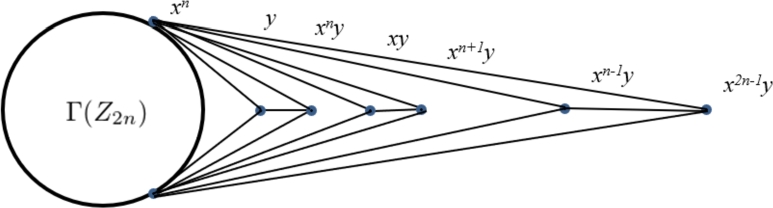
Γ(*Q*_4*n*_).

The graph of $\Gamma (Q_{4n})$ is given in Fig. 1, McKemmie (2017).

For i=0,1,…,n−1, we have $\langle x^{i}y\rangle =\{1,x^{i}y,x^{n},x^{n+i}\}=\langle x^{n+i}y\rangle$. The element $x^{i}y$ is adjacent only to $1,x^{n}$ and $x^{n+i}$ and the induced subgraph on $\left\{1,x^{n},x^{n+i}\right\}$ is complete. Therefore the elements $y,xy,x^{2}y,\ldots ,x^{2n-1}$ are extreme vertices of the group $\Gamma (Q_{4n})$. Use Theorem 5 and Corollary 2 to get the following characterization of the extreme vertices of $\Gamma (Q_{4n})$.

Theorem 17 *The extreme vertices of* $\Gamma (Q_{4n})$ *are* $y,xy,x^{2}y,\ldots ,x^{2n-1}y$ *if and only if n has two different odd prime factors. If* $n=2^{m}q^{k}$ *where q is an odd prime number and* m≥1 *, then the extreme vertices of* $\Gamma (Q_{4n})$ *are* $y,xy,x^{2}y,\ldots ,x^{2n-1}y$ *, all elements of* $Q_{4n}$ *of order* $2^{m+1}$ *and all elements of* $Q_{4n}$ *of order* $q^{k}$ *. If* $n=q^{k}$ *, then the extreme vertices of* $\Gamma (Q_{4n})$ *are* $y,xy,x^{2}y,\ldots ,x^{2n-1}y$ *and all elements of* $Q_{4n}$ *of order* $q^{k}$ *. If* $n=2^{m}$ *, then the extreme vertices of* $\Gamma (Q_{4n})$ *are all elements of* $Q_{4n}$ *except the identity and* $x^{n}$ *.*

## Declarations

### Author contribution statement

Omar A. AbuGhneim: Conceived and designed the analysis; Analyzed and interpreted the data; Wrote the paper. Mohammed Abudayah: Conceived and designed the analysis; Analyzed and interpreted the data; Contributed analysis tools or data; Wrote the paper.

### Funding statement

This research did not receive any specific grant from funding agencies in the public, commercial, or not-for-profit sectors.

### Data availability statement

Data will be made available on request.

### Declaration of interests statement

The authors declare no conflict of interest.

### Additional information

No additional information is available for this paper.
